# Vector diversity and malaria prevalence: global trends and local determinants

**DOI:** 10.1098/rspb.2025.2032

**Published:** 2025-11-26

**Authors:** Amber Gigi Hoi, Benjamin Gilbert, Nicole Mideo

**Affiliations:** ^1^ Department of Ecology and Evolutionary Biology, University of Toronto, Toronto, Ontario M5S 3B2, Canada

**Keywords:** Malaria Atlas Project, vector-borne disease, vector ecology, disease ecology, macroecology

## Abstract

Identifying determinants of global infectious disease burden is a central goal of disease ecology. While it is widely accepted that host diversity structures parasite diversity and disease prevalence, the influence of diversity in vectors—obligatory intermediate hosts for many parasites—has rarely been examined. Malaria, for instance, can be transmitted by over 70 mosquito species, but the impact of this diversity on malaria risk remains unclear. Further, environmental factors, like temperature, may modify this impact by influencing arthropod life history and behaviour. We studied the relationship between vector diversity, malaria prevalence and environmental attributes by curating and analysing data from open-access sources. Globally, the association between vector species richness and malaria prevalence differed by latitude, indicating strong dependence on environmental conditions. Processes by which the environment impacts vector community assemblage and function, and subsequently disease prevalence, varied across regions. In Africa, the environment exerted a top-down influence on disease by shaping vector communities, whereas in Southeast Asia, disease prevalence depended on more complex interactions between the physical and socioeconomic environment (rainfall and GDP) and vector diversity. This work highlights the role of vector diversity in structuring disease distribution and offers insights to disease macroecology theory.

## 1. Introduction

Global infectious disease burden remains high despite decades of control efforts [1]. New diseases are emerging at a rapid pace, and many existing diseases persist [2]. These threats to public and global health are not distributed evenly around the world but tend to be concentrated in the tropics [3,4]. Specifically, there is a clear latitudinal gradient of increasing infectious disease burden from the poles towards the equator, both in terms of disease prevalence [3] and the numbers of distinct diseases [4–6].

**Figure 1.**
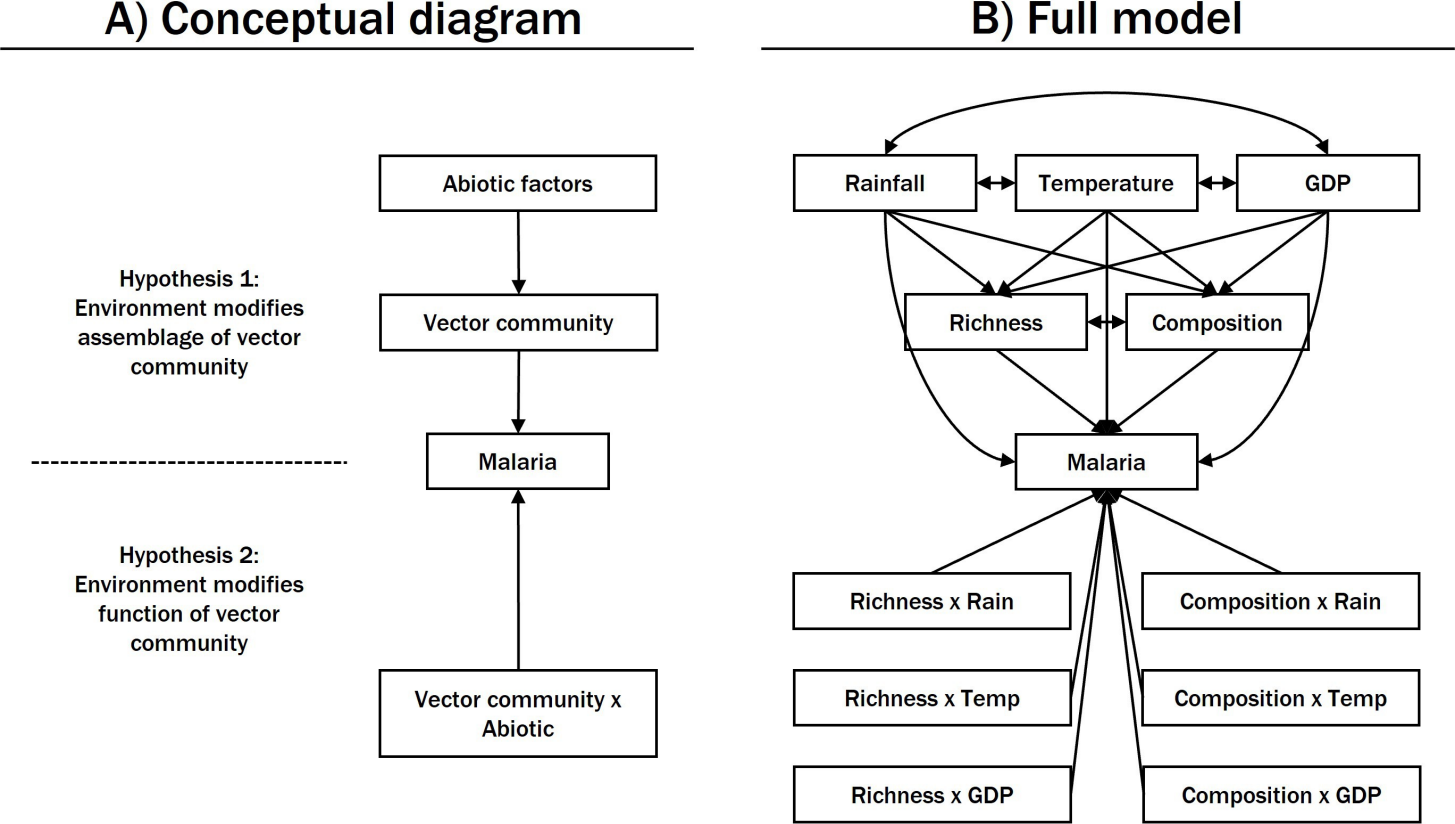
Path diagram representing hypothesized relationships between characteristics of the abiotic environment, vector community attributes and malaria prevalence. (A) The environment may influence disease risk via vector community assemblage (Hypothesis 1) or function (Hypothesis 2). (B) Path diagram for the hypothesized full model. Paths (i.e. arrows) represent the direction of the hypothesized causal relationships between variables, with arrows pointing from the predictor to the response. Double-headed arrows indicate hypothesized correlation between variables.

Ecologists have long been captivated by the latitudinal gradients in biological patterns. Much effort has been devoted to understanding such gradients in the biodiversity of terrestrial fauna and flora [7,8], which are hypothesized to be driven by environmental factors that covary with latitude. For instance, the tropics consist of environments conducive to supporting large and diverse host communities through high energy and water supply, coupled with high environmental heterogeneity (‘climatically based energy hypothesis’; [4]). More recently, the study of latitudinal gradients has extended to disease distributions [9,10], where global analyses found parasite diversity and prevalence tracked well-established gradients in mammalian and avian diversity [4,6,10]. One interpretation of these findings is that the effects of the environment on disease distributions are mediated through its influence on host community structure (figure 1A, top; [11]). Alternatively, the environment may induce changes in host or vector behaviour and life history, and subsequently their exposure to disease, independent of any effect on local community structure (figure 1A, bottom). For instance, how humans engage the outdoors may change depending on the weather (e.g. warm and sunny versus torrential downpour), thereby altering their exposure to disease vectors such as ticks and mosquitoes (e.g. [12,13]). Economic incentives may also drive host movement in and out of endemic regions on short timescales (e.g. migrant workers), sparking outbreaks and contributing to seasonal disease cycles [14]. While latitude remains a powerful proxy for capturing the myriad factors that covary along these clines to shape large-scale biological patterns, moving beyond latitude to elucidate the general ecological principles that contribute to patterns of disease is crucial to informing disease control policy and predicting future outbreaks.

At the macroecological scale, insect diversity conforms to a latitudinal diversity gradient [7,15], leading to the hypothesis that vector diversity promotes disease transmission similarly to host diversity [16,17]. Many parasites can be transmitted by multiple vector species, and vector communities are rarely homogeneous across space and time. Human malaria parasites, for example, predominantly infect just one vertebrate host in natural systems (occasionally also infecting other primates; [18]), but they can utilize more than 70 species of mosquitoes as vectors [19]. Historically, malaria research and control programmes have largely focused on a few vector species that are the main contributors to malaria transmission (‘dominant’ or primary vectors). Only recently has there been growing recognition of the contributions of other previously overlooked species (secondary vectors) to malaria transmission [20,21]. These mosquito species encompass substantial diversity in seasonal activity, habitat and feeding preferences [22], such that different assemblages of vectors should have differential impacts on transmission dynamics [23]. However, as far as we are aware, latitudinal gradients have not been investigated for the subset of mosquito species that are competent vectors—a distinction that is important for predicting how vector diversity should influence disease risk. Local analyses suggest that vector diversity amplifies malaria prevalence [23], but it is unclear whether this association is generalizable over large spatial scales and across environmental contexts in which vector–host interactions occur.

The impacts of environment on disease spread are particularly prominent in vector-borne disease systems since, as ectotherms, the vital and activity rates of arthropods are temperature-dependent [4]. Temperature-regulated life history traits also determine vector competency [24] and they scale up to influence vector population dynamics [25,26] and species distributions [27]. Many mathematical models of vector-borne disease transmission therefore leverage thermal performance curves to predict vector abundance and disease risk, and they have been applied to systems including Malaria, Dengue and Zika (see [26] for a recent review of such models). The broader influences of temperature (among other abiotic factors) on vectors at the community level, however, remain largely understudied. One of the fundamental questions is whether local environmental filters promote different mosquito vector community assemblages (figure 1A, top; [28]) or modify the disease transmission capacity of those communities (figure 1A, bottom; [29]). These two pathways have been demonstrated independently in tick-borne parasites of primates in Africa [11] and midge-borne viruses of wild ungulates in southeastern United States [29]. However, few studies have examined them in tandem or investigated their relative contribution to shaping disease patterns.

A clear vision of how biotic and abiotic drivers of disease intersect is an asset in combatting infectious diseases in the ever-changing Anthropocene [30]. This study seeks to bolster our current understanding of environmental effects on vector population dynamics and disease [26,31] to consider the influence of vector diversity and its interaction with environmental effects that may modify entomological risk. We build our analysis in multiple stages to capture both macroecological trends and general patterns in local disease drivers. First, we investigate whether global malaria vector diversity is consistent with expectations of a latitudinal gradient. Next, we determine whether vector species richness is associated with malaria prevalence, and whether this association is homogenous across latitudes. Our approach allows us to assess whether global patterns hold at smaller spatial scales or if the relationship between vector diversity and malaria prevalence is context-dependent. Finally, we adopt a structural equation modelling framework to elucidate pathways through which the environment influences malaria transmission in the major malaria-endemic regions of sub-Saharan Africa and South Asia, Southeast Asia, and Pacific Islands (hereafter Southeast Asia). Malaria is prevalent in sub-Saharan Africa, with young children suffering mild to severe symptoms and adults often having asymptomatic infections (i.e. holoendemic), whereas in Southeast Asia, malaria transmission is less stable and largely driven by seasonal outbreaks (i.e. hypoendemic) ([32,33]; table 1; electronic supplementary material, S1). These regions also broadly differ in climate and landscape, vector management and disease control strategies, as well as the biology of the hosts, vectors and parasites present ([32,33]; table 1; electronic supplementary material, S1). In light of these distinctions, we assess the influence of the environment on vector communities and malaria independently in each region.

**Table 1.** Epidemiological, biological, environmental and socioeconomic characteristics of the malaria transmission context in sub-Saharan Africa and Southeast Asia. In general, there are broad differences in the ecological and socioeconomical background of the two continents, as well as substantial intra-continental variation in these factors. We focused on the former with the aim of providing a foundation for our analysis plan and for the interpretation of our results.

characteristic	sub-Saharan Africa	Southeast Asia
epidemiology	holoendemic—malaria infections are prevalent, especially in young children [32]	hypoendemic—malaria transmission is unstable resulting in relatively few cases [32]
vector	dominated by a few human-specialist vectors [34]	communities tend to be more species rich and consist of more generalist vectors [35]
parasite	predominantly *P. falciparum* [32]	co-circulation of *P. falciparum* and *P. vivax*, occasional spillover of *P. knowlesi* [32]
climate	warmer, more humid and more homogeneous [33]	cooler, drier and more heterogeneous [33]
socioeconomics	historically considered a disease of the rural poor [36]	disease outbreaks often attributed to movement of large migrant worker populations [32]

## 2. Material and methods

### (a) Data sources and management

**Figure 2.**
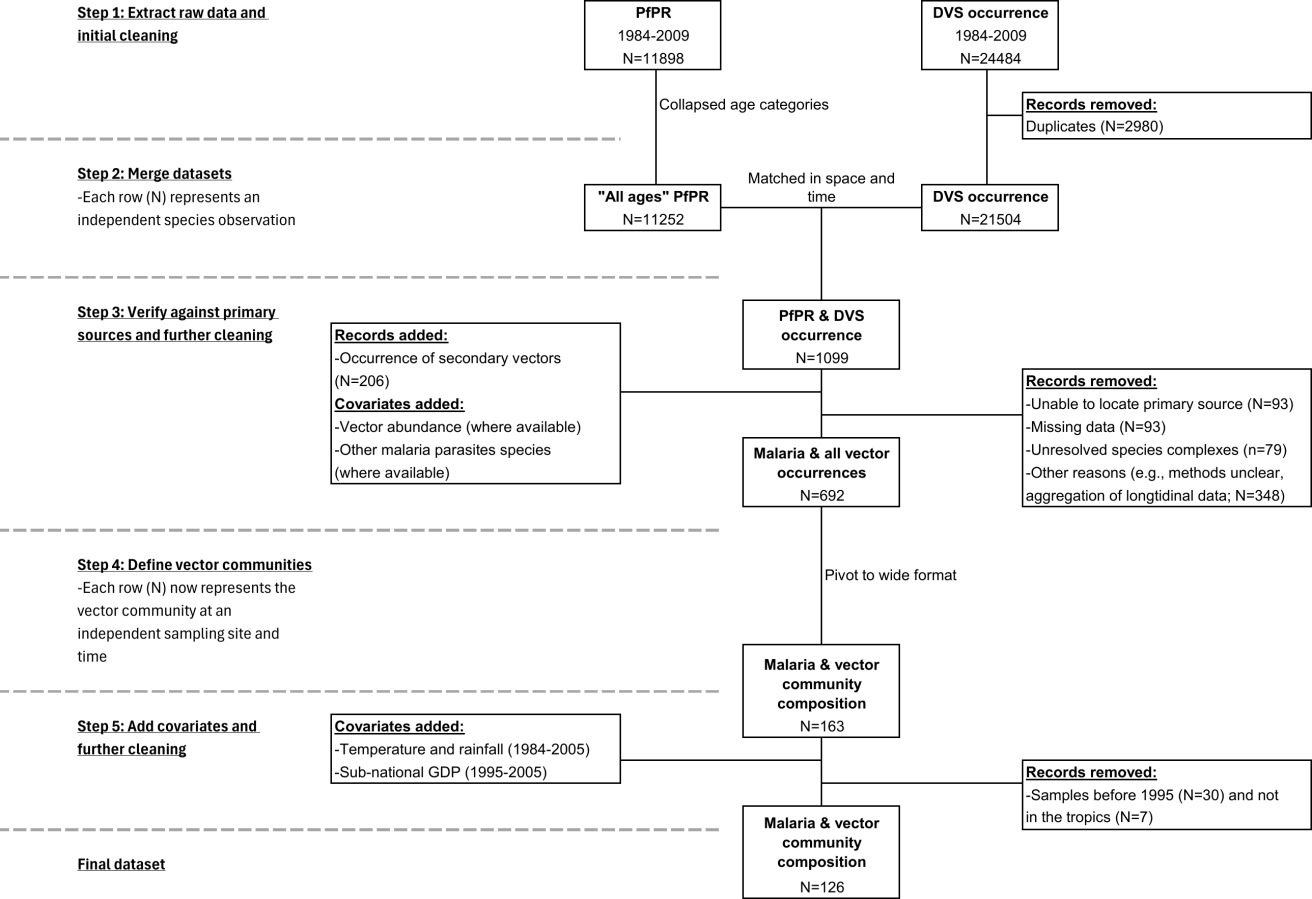
Flow chart of the data compilation pipeline. PfPR: *Plasmodium falciparum* parasite rate. DVS: dominant vector species. Details of data sources and cleaning procedure can be found in electronic supplementary material, S2.

We curated a dataset of global malaria prevalence and vector species distribution by integrating information from multiple open-access databases. The data cleaning procedure is depicted in figure 2 and detailed in electronic supplementary material, S2. Briefly, epidemiological (*Plasmodium falciparum* parasite rate) and entomological (occurrence of primary vector species) data were extracted from the Malaria Atlas Project (MAP; [37]) and were matched in space (in the same 1 × 1 km^2^ grid) and time (overlapped in study period). Primary data sources were reviewed vigorously to ensure accurate data entry and evaluated for suitability for inclusion to mitigate potential methodological bias in the data. Studies that did not conduct formal entomological surveys (e.g. targeted captures or anecdotal sightings, *n* = 3) or did not report all species caught (*n* = 19) were removed to ensure that the observed diversity reflect true diversity as closely as possible. Data from sites with active disease control intervention (e.g. bednet trials, *n* = 7 studies) were also removed, while data from control sites or pre-treatment values were retained when available. Information on vector abundance, presence of secondary vectors, and other *Plasmodium* species were extracted from primary sources when available. Many vector species belong to complexes or groups that are not always taxonomically resolved; we recorded these entries at the lowest reported classification level available (except for the *An. gambiae* complex, where records that had not been resolved were excluded; *n* = 79). The validated vector species occurrence dataset was then aggregated to the community level, yielding 163 independent characterizations of vector community composition and malaria prevalence from 112 locations globally.

Monthly temperature and precipitation were extracted from Copernicus Climate Change Service [38]. The sub-national (i.e. administrative units within a country) per capita gross domestic product (hereafter GDP), used as our proxy for economic condition, was extracted from Kummu *et al.* [39] and was only available from 1990 to 2005. Epidemiological and entomological data that fell outside of this time span (1983–1989, *n* = 30) were thus removed. We also removed surveys that took place outside of the tropics (7 out of 133) as most observations were made within.

The final dataset therefore consisted of 126 independent surveys of vector communities and malaria prevalence from 86 locations. Complete species assemblages (i.e. a list of all species present) were available for all surveys, and species relative abundance was available for 83 surveys. We used species richness (number of species) as the primary measure of vector diversity and the proportion of species that were primary vectors (number of primary vector species/total number of species) as a proxy for vector community composition to capture the effect of the presence of secondary vectors, even if rare. Although species relative abundance data was available for a subset of surveys, variation in sampling protocol across studies (electronic supplementary material, S1) precluded standardization and meaningful comparison [40,41], and so we only used these data for descriptive purposes and not in our main statistical analysis.

### (b) Statistical analysis

We first explored global mosquito diversity and malaria prevalence patterns by mapping these attributes (*n* = 126), and examined the representation of primary vectors in communities using the subset of data where species abundances were available (*n* = 83). We then used generalized linear mixed models (GLMM) to test for associations between vector species richness and malaria prevalence. In total, we constructed four models that included the effects of primary or all vector species richness, with or without a latitude by species richness interaction, in a factorial manner. This setup allowed us to investigate the following two questions: (i) is the effect of vector species richness predominantly driven by primary vectors, or does inclusion of secondary vectors better explain malaria prevalence (by contrasting models with primary versus total vector species richness), and (ii) is the effect of vector species richness general, or does it depend on the latitude, as a proxy pf the environment (by contrasting models with and without latitude by vector species richness interaction)? Lastly, we developed structural equation models (SEMs) to dissect the environmental factors that influenced vector diversity and disease relationships. Malaria prevalence, total vector species richness, and vector community composition (proportion of species that were primary vectors) were included as response variables. Time-matched temperature, rainfall and GDP, and their interactions with vector species richness and community composition, were included as predictors. The SEM was designed to distinguish between two hypothesized pathways through which the environment may influence malaria transmission: mediated through vector community structure or interacting with vector community function (figure 1). This approach allowed us to explicitly account for the potential associations between factors that may otherwise pose issues of multicollinearity in simple regressions, but is prone to overfitting due to our relatively small sample size. We therefore performed model selection to reduce the number of variables in the SEM. Technical details of the GLMMs and SEMs can be found in electronic supplementary material, S3 and S4, respectively.

Datasets assembled from diverse sources, methodologies, regions and time periods are inherently challenging to analyse due to the numerous sources of variation involved. Geographic differences in malaria epidemiology and vector sampling methodology as a result of local vector diversity or abiotic conditions could bias the estimation of disease-diversity relationships. Over the study period, there were also increasing efforts to control malaria through enhanced vector management efforts and improved treatment availability and efficacy. This could potentially obscure the relationship between vector diversity and malaria prevalence, making it more difficult to detect. To account for these sources of variation, we included ‘year’, ‘country’ and ‘site’ as random effects in all statistical models. We also partitioned our data by region and fitted the SEM to sites in Africa (*n* = 64) and Asia (*n* = 57) separately. The remaining data points from South America (*n* = 5) were excluded from this analysis.

## 3. Results

### (a) Overview of data

**Figure 3.**
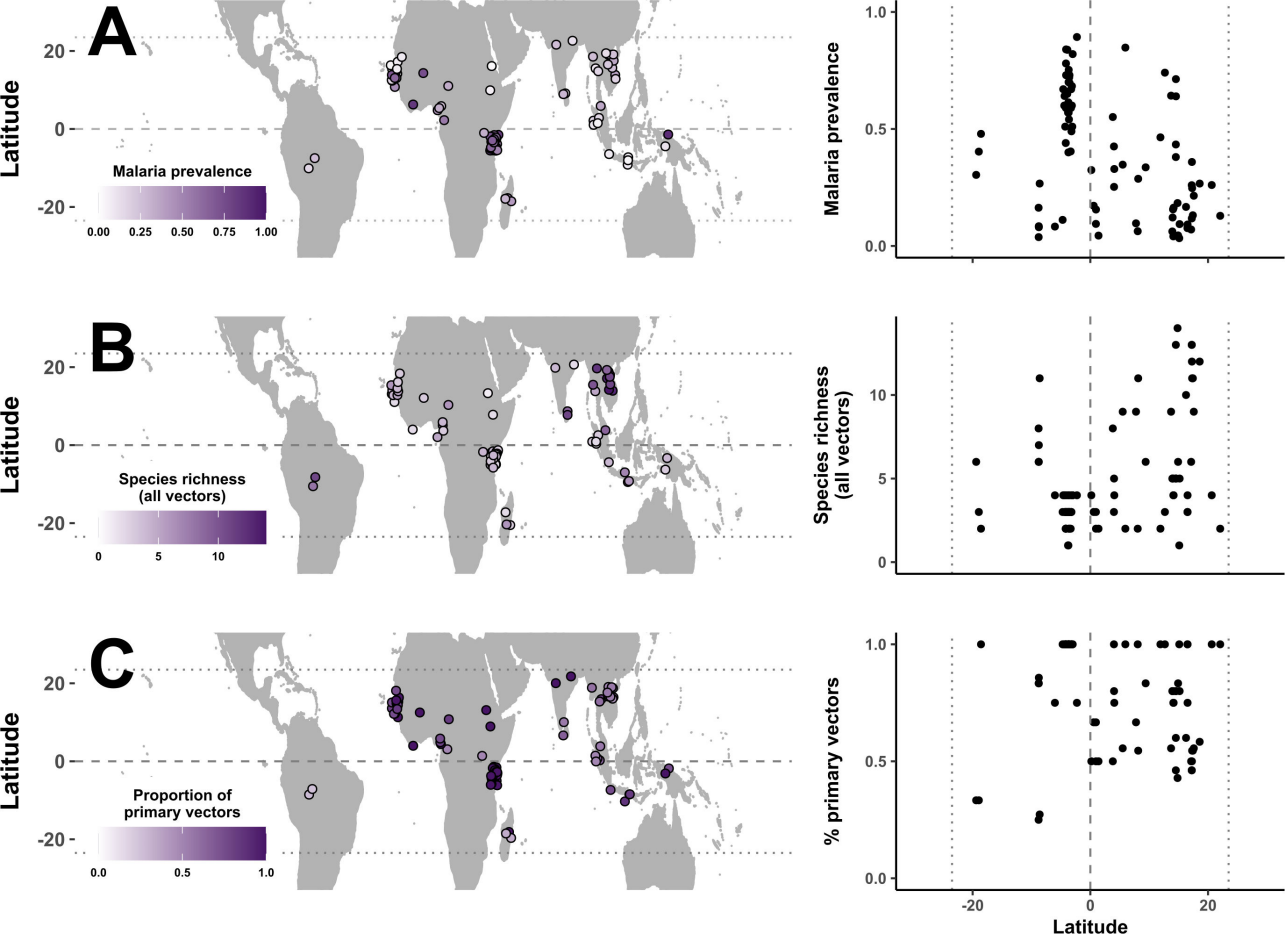
Distribution of (A) malaria prevalence, (B) total vector species richness and (C) percent primary vectors by species richness across the tropics, 1990–2005. Each data point on maps and scatterplots represents one site (*n* = 86). For sites that were sampled repeatedly over time, the average malaria prevalence and total number of vector species observed over the study period was presented. Dashed line represents the equator, and dotted line marks the tropical region (latitude range 23.5°S to 23.5°N) in all plots.

The final dataset consisted of 126 independent descriptions of vector community composition from 86 locations across three continents, 17 countries and 15 years (1990–2005). Malaria prevalence was highest in sub-Saharan Africa (figure 3A). Out of 54 globally recognized primary vectors of malaria [19,42], 30 occurred in our dataset. An additional 30 secondary vector species were identified and added to the dataset from our review of primary sources. A list of all vector species included in this study can be found in electronic supplementary material, S5. Sub-Saharan Africa, Asia and South America did not share any vector species in the time frame included in this study. Sites in Asia were more species rich (figure 3B) with a higher proportion of species being secondary vectors (figure 3C). This observation may, in part, reflect regional differences in sampling methodology: surveys in Asia predominantly employed mixed sampling approaches (91%), whereas those in sub-Saharan Africa tend to sample indoors (64%), potentially under-detecting secondary vectors. Other regional differences include lower temperature and humidity, and higher per capita GDP at Asian sites compared with those in Africa. Detailed information on environmental variables (temperature, rainfall, GDP), vector community attributes (species richness, composition), malaria prevalence, and vector sampling methodology across the two regions are presented in electronic supplementary material, S1.

### (b) Global trends in vector diversity and malaria burden

Malaria prevalence was negatively correlated with distance from the equator (Pearson’s correlation = −0.43, *p* < 0.01; figure 3A), however, vector diversity did not conform to this predicted latitudinal gradient. Vector species richness was positively associated with distance from equator (Pearson’s correlation = 0.49, *p* < 0.01; figure 3B), though more primary vectors were observed in the northern hemisphere (figure 3C), suggesting that vector diversity in the southern sites was driven by the presence of secondary vectors.

**Figure 4.**
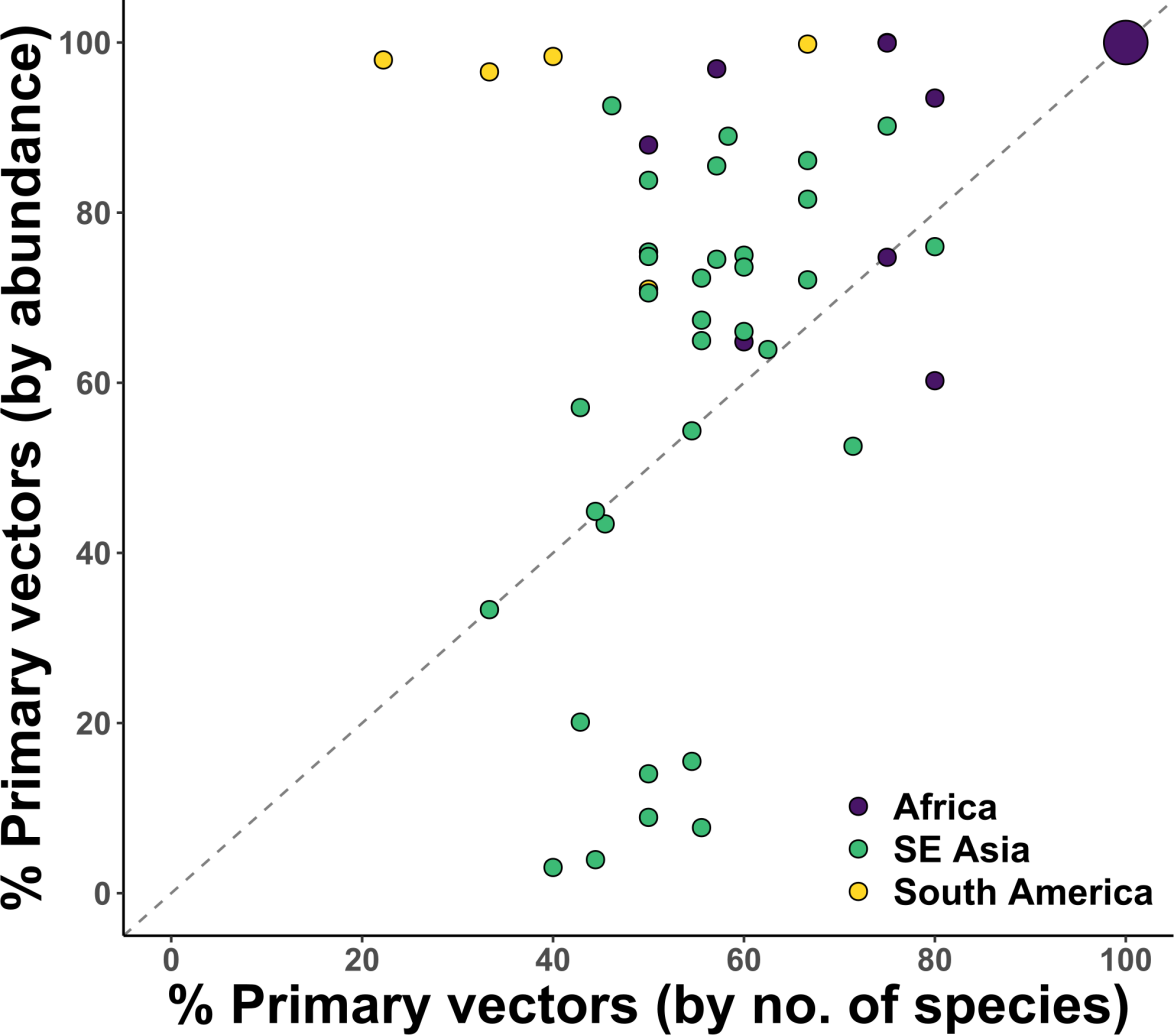
Representation of primary vectors in each community by species richness and abundance relative to all vectors, for subset of data where abundance was available (*n* = 83). Each data point represents one observation at the site level, except for the large circle on the top-right corner which represents 34 vector communities in sub-Saharan Africa composed entirely of primary vectors. Dashed line represents a one-to-one relationship between the relative richness and relative abundance of primary vectors. Sites were separated into regions based on continent.

Further examinations of the subset of observations with relative abundance data (*n* = 83) revealed that primary vectors tend to be over-represented in abundance relative to their species richness (figure 4). Thirty-four vector communities (located predominantly in east Africa) consisted entirely of primary vectors, and most of the other communities aggregated above the 1:1 line in figure 4, indicating that their species compositions were not even, but rather dominated by primary vectors. One interpretation of this pattern is that vector competency is positively correlated with competitive ability. Alternatively, it may reflect the fact that being numerically dominant is a key criteria used in the incrimination of primary vectors, regardless of intrinsic competence [19], or be an artefact of common vector sampling techniques favouring primary vectors by design. The main exceptions to this observed pattern were several sites in Laos (average distance between sites was 62.6 km) in which primary vectors existed in relatively low abundances despite making up approximately 50% of vector species.

### (c) Latitudinal gradient in vector diversity–disease relationships

**Figure 5.**
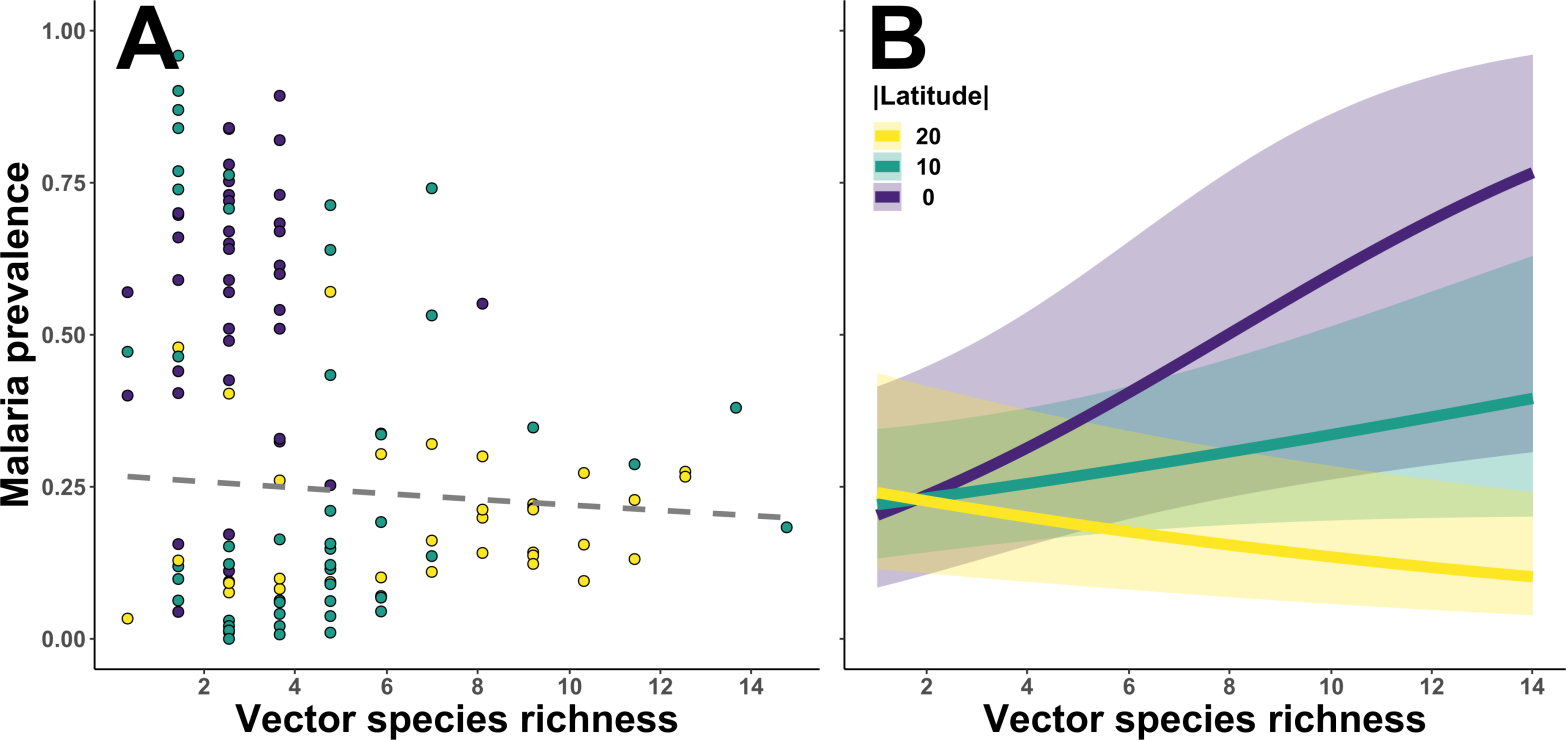
The relationship between malaria prevalence and total vector species richness in the tropics. (A) The relationship between malaria prevalence and vector species richness was not statistically significant (grey dashed line). Each data point represents one independent observation (*n* = 126), and the colour corresponds to the latitudinal position of the site rounded to the nearest 10 degrees. (B) There was a statistically significant interaction between vector species richness and distance from equator (absolute value of latitude). Lines show predicted effects on malaria prevalence and bands represent 95% confidence interval of prevalence estimates. Details of model structure and results for both assessed relationships can be found in table 2 and the main text.

We used generalized linear mixed models (GLMMs) to examine the relationship between vector diversity and malaria prevalence at study sites located within the tropics (*n* = 126). This relationship was strongly dependent on latitude, a proxy for environmental conditions known to covary along this gradient (table 2, figure 5). The apparent negative association between vector species richness (both primary and total species richness) and malaria prevalence was not statistically significant (table 2, figure 5A), however, vector species richness interacted with latitude to influence malaria prevalence (table 2, figure 5B). Specifically, there was a strong positive association between vector species richness and malaria prevalence near the equator, but this relationship weakens and reverses moving towards the edge of the tropics. These effects were weak (fixed effects explained approximately 1% of variation in the data for both models; table 2) but were captured despite the vast variation between countries and sites and over time (fixed and random effects together explained more than 30% of the variation in the data for both models; table 2, electronic supplementary material, S6).

**Table 2.** Generalized linear mixed models for malaria prevalence at 126 sites across the tropics. The response variable for all models was malaria prevalence. All predictors were centred and scaled to unit variance to facilitate model convergence. Several random effects were included to account for non-independence in the data: site (some sites were measured repeatedly), country and year, nested in that order. β: regression coefficients. SE: standard error. DF: degrees of freedom. AIC: Akaike information criterion. Conditional R^2^: proportion of variation explained by fixed and random effects combined. Marginal R^2^: proportion of variation explained by fixed effects only. R^2^ statistics were estimated using methods outlined in [43,44].

	primary vectors only				all vectors			
	w/o interaction		w/ interaction		w/o interaction		w/ interaction	
predictors	*β*	SE	*β*	SE	*β*	SE	*β*	SE
intercept	−1.13***	0.30	−1.03***	0.29	−1.12***	0.30	−1.05***	0.30
species richness	−0.001	0.04	0.14*	0.06	−0.08	0.06	0.19	0.12
|latitude|			−0.23	0.21			−0.23	0.22
species richness * |latitude|			−0.16**	0.06			−0.26**	0.10

variance components	var	SD	var	SD	var	SD	var	SD
year	0.88	0.93	0.71	0.84	0.84	0.92	0.70	0.83
year(country)	1.03	1.02	1.13	1.06	1.07	1.04	1.25	1.12
year(country(site))	0.31	0.55	0.30	0.55	0.31	0.56	0.30	0.55
DF	121		119		121			119
AIC	1299.2		1293.9		1297.1			1293.5
conditional R^2^	31.96 %		32.07%		32.39%			33.09%
marginal R^2^	0%		0.93%		0.1%			0.90%

*p < 0.05, **p < 0.01, ***p < 0.001

### (d) Structural equation models revealed regional differences in drivers of malaria burden

Our SEMs decompose latitude into underlying abiotic factors known to influence species distribution and interactions, and were formulated to compare two hypotheses about how these factors were associated with malaria prevalence (figure 1A). As such, fixed-effect predictors now capture a higher proportion of total variance in malaria prevalence explained compared with GLMMs (tables 2 and 3, electronic supplementary material, S6), and we were able to discern regional differences in factors shaping vector communities and influencing malaria prevalence.

**Table 3.** Results for structural equation models fitted to regional data (sub-Saharan Africa and Southeast Asia), following model selection. All sub-models include a random effect term to account for non-independence in the data: site (some sites were measured repeatedly), country and year, nested in that order. A blank cell indicates a path that was dropped during model selection (via procedure detailed in main text). Raw effect estimates (Raw β), standardized effect estimates (Std β), and raw standard errors (SE) are provided. The d-separation test (H_0_: no important paths missing in the model; [45]) was used to assess model fit, and the associated Fisher’s C statistic, degrees of freedom and *p*-value are reported. Conditional R^2^ (R^2^c): proportion of variation explained by fixed and random effects combined. Marginal R^2^ (R^2^m): proportion of variation explained by fixed effects only. R^2^ statistics were estimated using methods outlined in [43,44].

		Africa only			Asia only		
response	predictor	Raw β	std β	SE	Raw β	std β	SE
malaria prevalence	species richness	0.26*	0.17*	0.11	1.47***	2.28***	0.42
	composition	3.06**	0.23**	1.15	−8.61	−0.75	7.47
	rainfall				0.30	0.14	0.50
	temperature	0.05	0.01	1.68	−0.07	−0.04	0.06
	GDP				1.32	0.17	2.28
	richness * rain				0.10**	0.33**	0.03
	richness * temp	−0.14	−0.13	0.15			
	richness * GDP				−0.44***	−2.21***	0.13
	composition * rain				−1.35*	−0.44*	0.53
	composition * temp	1.48	0.35	1.49			
	composition * GDP				2.95	0.93	2.30
species richness	rainfall	−0.37**	−0.74**	0.14			
	temperature	0.14	0.20	0.17			
	GDP	0.47	0.18	0.62	−0.63	−0.37	0.44
composition	rainfall						
	temperature						
	GDP						
**correlations**							
species richness	composition	−0.45***			−0.25*		
temperature	rainfall	0.72***			0.10		
GDP	temperature	0.62***			−0.37**		
GDP	rainfall	0.71***			0.02		
**goodness of fit**							
Fisher’s C		1.39			1.09		
degrees of freedom		4			4		
*p*‐value		0.85			0.90		
malaria R^2^c		40.14%			33.88%		
malaria R^2^m		9.71%			6.95%		
species richness R^2^c		16.99%^a^			57.37%		
species richness R^2^m		16.99%^a^			6.91%		

*p* < 0.05, **p < 0.01, ***p < 0.001

^a^ Random effects explained ~0.000000001% of the variance in the response variable.

**Figure 6.**
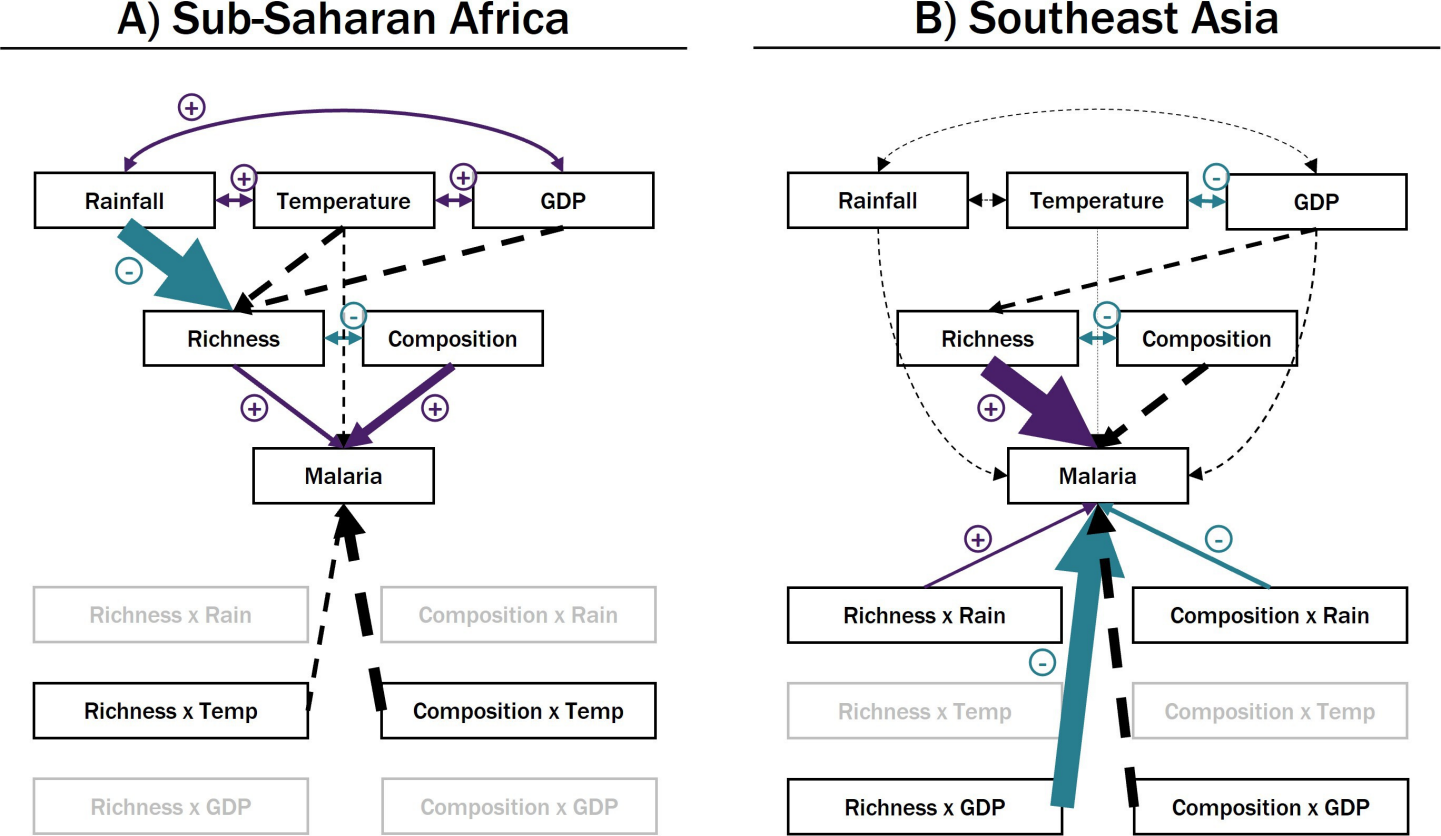
Path diagrams depicting model selection and fit results for (A) sub-Saharan Africa (*n* = 64) and (B) Southeast Asia (*n* = 57). Variables in grey were dropped during model selection (procedure detailed in the main text). Paths (i.e. arrows) represent the direction of the modelled causal relationship between variables, with the arrow pointing from the predictor to the response. Solid arrows are associations that were statistically significant while dashed arrows were not; positive associations are in purple and negative associations are in teal. Arrow size is proportional to the strength of paths within each model but not between models. Double-headed arrows indicate correlations and are not scaled to effect size. See table 3 and electronic supplementary material, S7 for detailed results.

In sub-Saharan Africa, malaria prevalence was positively associated with both vector species richness and proportion of primary vector species (table 3, figure 6A, electronic supplementary material, S7). Since vector species richness was negatively correlated with the proportion of primary vector species in a community, the positive effect of species richness on malaria thus diminishes with the introduction of secondary vectors in species-rich communities. The impact of species richness could also have been over-estimated due to the under-detection of secondary vectors. Rainfall, temperature and GDP covaried, but rainfall was the only environmental factor directly associated (negatively) with vector species richness. Taken together, sites with more rain, warmer temperatures and higher GDP tended to house depauperate mosquito communities consisting mostly of primary vectors. The net effect in these sites is a lower malaria prevalence. Most of the interaction terms between biotic and abiotic factors were dropped during model trimming, and the ones retained in the final model (interactions with temperature) were not significant predictors of malaria. This suggested that in sub-Saharan Africa, the environment influenced malaria prevalence in a top-down manner by structuring local vector communities (figure 1A, Hypothesis 1).

Results were drastically different in Southeast Asia. Although species richness still had a significant positive association with malaria prevalence, none of the environmental variables were associated with vector community attributes (table 3, figure 6B). Rather, the environment influenced malaria by altering the effects of vector diversity (vector community × abiotic interactions). Rainfall modified the relationship between vector species richness, composition and malaria: increasing rainfall tipped a negative association between vector species richness and malaria to being slightly positive and dampened the positive association between composition and malaria (figure S7.2 in electronic supplementary material, S7). Similarly, the negative interaction between species richness and GDP meant that the slight positive effect of species richness on malaria weakens and reverses in sites with higher economic output (figure S7.2 in electronic supplementary material, S7). The overall significance of vector community × abiotic interactions in influencing malaria prevalence in Asia suggested that the predominant effect of the environment, especially that of the socioeconomic environment, was to alter the function of vector communities (figure 1A, Hypothesis 2).

## 4. Discussion

Recent years have seen great progress toward understanding processes that shape infectious disease patterns around the world [9,10]. The role of mammalian and avian diversity has been the focus of many such studies, as these taxa commonly serve as alternative hosts to human parasites. For vector-borne diseases such as malaria, vector diversity, which was expected to conform to the same latitudinal gradient of diversity observed for most terrestrial fauna, has long been hypothesized to influence disease distribution in the same manner as their vertebrate host counterparts [16,17]. Yet, these associations have not been tested and global vector diversity has not been fully mapped. While tremendous efforts have been made to map and predict the presence of individual vector species based on climate and habitat suitability and to understand the influence of vector population dynamics on disease transmission (e.g. [19]), now is the time to extend these approaches to consider vector communities more comprehensively.

In this study, we showed that vector diversity contributed to structuring global malaria distribution and found these relationships to be highly context-dependent. We uncovered vector distribution patterns that were not consistent with the assumed latitudinal gradient of diversity (figure 3B) and not aligned with that for the family *Culicidae,* nor for *Anopheles,* the only genus of mosquito with species that act as malaria vectors [15]. We demonstrate that the global vector species richness and malaria relationship (a negative trend that was not statistically significant; figure 5A) is not representative of local trends (a positive association that weakened with increasing latitude; figure 5B)—a case of Simpson’s Paradox [46]. Our SEM analysis further revealed important differences in environmen–diversity–disease relationships emerging from distinct transmission contexts across two malaria endemic regions: sub-Saharan Africa (table 3, figure 6A) and Southeast Asia (table 3, figure 6B). These insights demonstrate the salience of vectors in disease macroecology and offer a framework for advancing beyond species distribution by incorporating community assemblage and function under different environmental contexts.

The dataset we compiled showed that malaria vector species richness decreases from the edge of the tropics towards the equator (per site-level records), which is unexpected given the large number of *Anopheles* species apparently found near the equator (per country-level records from [15]). This poses the intriguing question of why those Anophelines near the equator are not considered vectors. Alternatively, this pattern may arise if low latitude areas were under-represented in our dataset, but that is not the case (figure 3). It also seems unlikely that malaria vectors would be systematically under-sampled compared with non-vector Anophelines, although the emphasis on primary vectors near human settlements may bias surveillance efforts, leading to the under-detection of secondary vectors. If there are in fact reservoirs of *Anopheles* mosquitos of unknown vector competency residing outside of sampling hotspots, then this should be a cause for concern [47,48]. Nonetheless, the observation of few competent vectors at low latitudes led to the inference of a negative association between vector species richness and malaria prevalence at the global scale (figure 5A). When this relationship was broken down by latitude, however, we found that vector species richness was in fact positively associated with malaria prevalence near the equator, and the effect weakened and reversed moving towards the edge of the tropics (figure 5B). These patterns remain quantitatively similar regardless of whether secondary vectors were included in the model (table 2), suggesting that the negative association at higher-latitude sites was not simply driven by the occurrence of less-competent species. Rather, this is a clear signal of context-dependency in vector diversity and disease relationships.

The two major malaria endemic regions in the world—sub-Saharan Africa and Southeast Asia—are known to differ broadly in malaria epidemiology, vector community structure and underlying disease transmission contexts [32,33]. Of particular relevance to this study were the differences in vector assemblages: their membership did not overlap, with sub-Saharan African vector communities dominated by a few human-specialist species (in terms of number of species), and Southeast Asian vector communities tending to be made up of generalist and opportunistic vectors (figure 4). Despite potential sampling bias, especially at sub-Saharan African sites, this pattern is consistent with that reported in the literature [32,33]. While vector species richness was positively associated with malaria prevalence in both regions, vector community composition only had an effect in sub-Saharan Africa (figure 6). One explanation of this pattern is that competence among vectors is much more even in Southeast Asia than in sub-Saharan Africa. Thus, the disease transmission capacity of vector communities would depend on the number of species present rather than their identities in Southeast Asia, but would be more strongly influenced by the representation of primary vectors in sub-Saharan Africa. These community-level characteristics probably also contribute to the observed variation in how vectors functioned in their respective environments. In sub-Saharan Africa, the environment exhibited a top-down influence on malaria prevalence via vector community structure, whereas in Southeast Asia, the environment instead modulated the disease transmission efficacy of vector communities. Considering that human specialist vectors mostly blood-feed and rest indoors, it follows that the environment would have less effect on their blood-feeding behaviour and success, but would nonetheless act as a filter for species occurrences. By contrast, in Southeast Asia, where vector communities consist mostly of generalists that blood-feed outdoors, their interactions with human hosts are much more likely to be modified by the ambient environment [49].

Climate change, along with many human activities and interventions such as land development and vector management programmes, have the potential to alter vector community structure [28,50]. For instance, urbanization often leads to landscape homogenization, which can shift vector community composition in favour of species that are well-adapted to those habitats. These changes are often idiosyncratic and depend on exactly how the niche was altered, as well as which species were available for recolonization from the broader regional pool [51]. Global vector species distribution maps have therefore been important assets for forecasting future disease risks [19]. In this current collection, we extended the MAP database [37] by adding in observations of secondary vectors when these were present in the original studies compiled in MAP. However, we do not have any information on surveys where only secondary vectors were found—these were omitted from MAP by design—and most country-level records (as used in [15]) are compiled at spatial and temporal scales that cannot be compared with MAP data (see [52] for a notable exception).

Improving global vector and vector-borne disease surveillance capacity, in particular, to establish long-term standardized monitoring programmes and to extend sampling to non-conventional locations [47,48], is key to making continued progress in disease control and outbreak management [53]. A solid understanding of vector behavioural ecology and precise definition of vector competence, especially that of secondary vectors, is also essential to further dissecting the associations between environment, vector communities and malaria transmission uncovered here. In this study, we were only able to distinguish vectors by their primary versus secondary vector designation and defined community structure as the proportion of species that are primary vectors. Ideally, we would use a trait-based definition of vector competence and standardized species abundance data to characterize vector communities by their community-level disease transmission capacity. Further information on how traits associated with competence are linked with those that determine phenology and habitat use would be essential for accurate predictions of how vector distributions and disease risk will shift when climate and habitat inevitably change. The future of malaria is being shaped by rapid environmental and societal change. An interdisciplinary and holistic approach is crucial to continuing to make progress in the race to elimination.

## Data Availability

Data and analysis script used for this study can be found on the Zenodo Repository [54]. Supplementary material is available online [55].
